# Steroid 5 alpha-reductase 3 (SRD5A3) promotes tumor growth and predicts poor survival of human hepatocellular carcinoma (HCC)

**DOI:** 10.18632/aging.104142

**Published:** 2020-11-20

**Authors:** Qicong Mai, Dafeng Sheng, Chengcong Chen, Qing Gou, Meng Chen, Xiaoting Huang, Heng Yin, Xiaoming Chen, Zide Chen

**Affiliations:** 1 Department of Interventional Radiology, Cancer Center, Guangdong Provincial People’s Hospital, Guangdong Academy of Medical Sciences, Guangzhou 510080, Guangdong, China; 2 PET/CT Center, Shantou Central Hospital, Affiliated Shantou Hospital of Sun Yat-Sen University, Shantou 515041, Guangdong, China; 3 Department of Radiation Oncology, Affiliated Cancer Hospital & Institute of Guangzhou Medical University, Guangzhou 510095, Guangdong, China; 4 Department of Medical Biotechnology, School of Basic Medical Sciences, Guangzhou University of Chinese Medicine, Guangzhou 510006, Guangdong, China; 5 The First School of Clinical Medicine, Southern Medical University, Guangzhou 510515, Guangdong, China

**Keywords:** steroid 5 alpha-reductase 3, hepatocellular carcinoma, proliferation, bioinformatics analysis, clinical significance

## Abstract

Steroid 5 alpha-reductase 3 (SRD5A3) is an important molecule in glycosylation metabolism and steroid hormone formation. It is differentially expressed in human fetal liver, endometrial cancer and prostate cancer; however, its prognostic value and biological function in hepatocellular carcinoma (HCC) remain unclear. Here, bioinformatics analysis was employed to explore the expression and prognostic significance of SRD5A3 in various cancers including HCC. Additionally, clinical specimens of HCC were applied to analyze the expression of SRD5A3. SRD5A3-underexpressed HCC cell lines were established to test the effect of SRD5A3 on cell proliferation in *in vitro* and *in vivo*. We found that the elevated expression of SRD5A3 was common in many cancers with poor prognosis. Moreover, public datasets and our specimens revealed that SRD5A3 was also upregulated in HCC tissues and associated with clinical stage and patient’s gender. Kaplan-Meier survival analysis showed that higher SRD5A3 level predicted poor overall survival, progression-free survival, relapse-free survival and disease specific survival in HCC patients. Further experiments showed that the lack of SRD5A3 inhibited the growth of HCC. Collectively, these findings indicate that SRD5A3 functions as an oncogene and might serve as a potential biomarker for prognosis and a therapeutic target for HCC.

## INTRODUCTION

According to the Global Cancer Statistics in 2018, liver cancer was the sixth most common and the fourth leading malignant tumor in the world [1]. About 841,000 new liver cancers and 782,000 liver cancer-related deaths occur annually [1]. Clinically, 75%-85% of primary liver cancers are pathologically diagnosed as hepatocellular carcinoma (HCC), which is considered to be induced by risk factors such as viral hepatitis B and C, alcohol, cirrhosis, fatty liver disease, diabetes, aflatoxin and aristolochic acid [1, 2]. Although radical surgery (such as hepatectomy, local ablation and liver transplantation) and palliative treatment (such as transarterial chemoembolization (TACE), molecular targeted therapy, and immunotherapy) are commonly used to treat patients with HCC, HCC metastasis and relapse are still prone to occur and eventually lead to an unsatisfactory prognosis [2–4]. The malignant behaviors of HCC are most likely due to the accumulation of changes in the genome and multifaceted molecular pathways [5, 6]. Therefore, the identification of molecules essential for the proliferation and progression of HCC is important, not only for predicting prognosis and targeted therapy, but also for a comprehensive understanding of molecular biological mechanisms involved in the development of HCC.

Steroid 5 alpha-reductase 3 (SRD5A3) is a protein-coding gene belonging to the steroid 5-alpha reductase family, which also includes steroid 5 alpha-reductase 1 (SRD5A1) and steroid 5 alpha-reductase 2 (SRD5A2) [7, 8]. These steroid 5-alpha reductase enzymes regulate the production of steroid hormones and male sexual development by catalyzing the conversion of testosterone into the most potent natural androgen 5 alpha-dihydrotestosterone (DHT) [9, 10]. Additionally, they are also involved in the activation of the androgen-androgen receptor (AR) pathway [9, 10]. Among them, SRD5A1 and SRD5A2 were reported to be highly expressed in human liver, and the protein level of SRD5A1 increased with the severity of nonalcoholic fatty liver disease (NAFLD) [11]. Moreover, the absence of SRD5A1 accelerated the progression of hepatic steatosis but protected against the development of NAFLD-related HCC [11]. Further research showed that the expression of SRD5A1 can be elevated by hepatocyte growth factor (HGF) through up-regulating the transcription factor Egr-1 [12]. In addition, SRD5A2 was found to be aberrantly methylated in HCC tissues (20 cases) but not in any corresponding non-HCC liver tissues (20 cases), and served as a biomarker for early diagnosis of HCC [13, 14]. All these studies indicated that SRD5A1 and SRD5A2 are involved in the carcinogenesis and development of HCC. As an isoenzyme of SRD5A1 and SRD5A2, the discovery of SRD5A3 was relatively late [15]. Recent studies demonstrated that SRD5A3 was overexpressed in human fetal liver, endometrial cancer, hormone-refractory prostate cancer as well as metastatic prostate cancer [10, 15–17]. However, SRD5A3 expression, its impact on the prognosis of HCC patients, and its role in the biological behavior of HCC have not yet been elucidated.

In the current study, SRD5A3 expression in different cancers including HCC was analyzed by bioinformatics, and further verified with clinical specimens of HCC tumor (HCC) and corresponding adjacent non-HCC liver (peri-HCC). Then, the correlation between SRD5A3 expression, clinical progression of HCC and survival prognosis of different cancer patients were studied. Furthermore, we examined the biological effects of SRD5A3 on HCC growth through *in vitro* and *in vivo* experiments. Finally, the molecular mechanisms behind these biological functions were explored.

## RESULTS

### SRD5A3 is highly expressed in various tumor tissues including HCC

**Figure 1:**
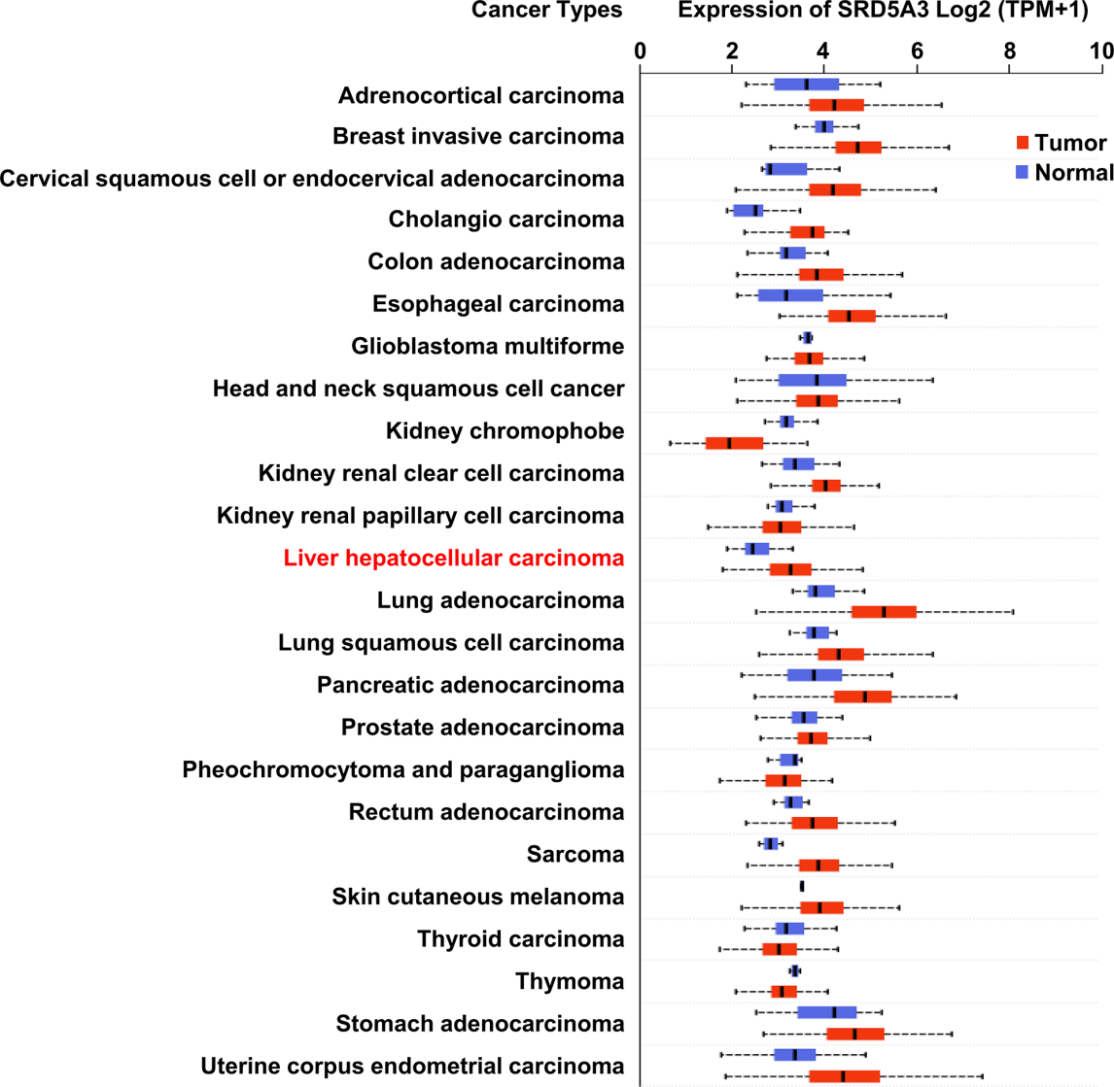
**The expression of SRD5A3 in various carcinomas.** Transcriptional expression of SRD5A3 in 24 types of cancer (the UALCAN web portal).

**Figure 2:**
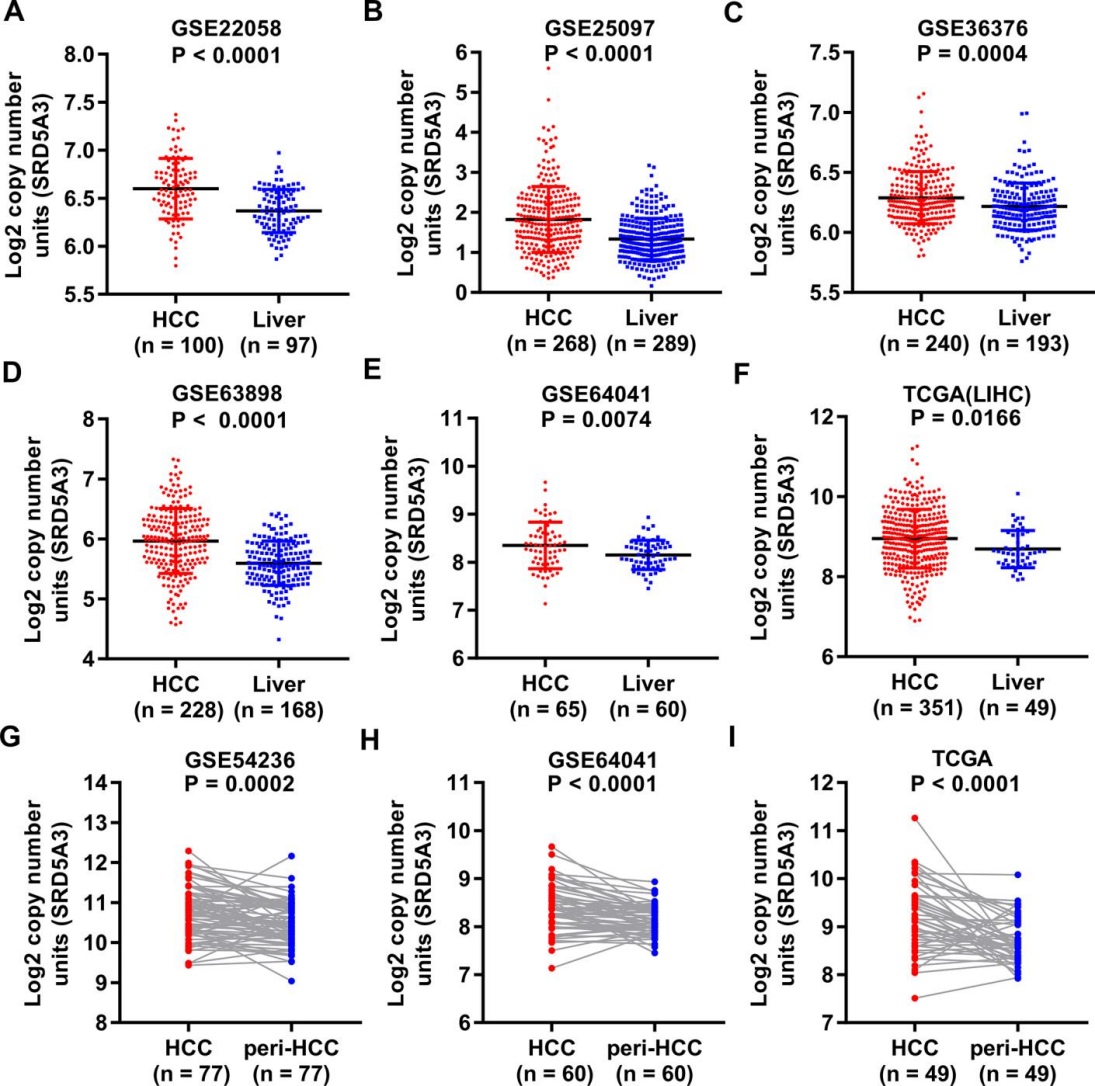
**SRD5A3 is generally highly expressed in HCC tumor tissues compared to non-HCC liver tissues.** SRD5A3 expression in HCC tumor tissues and non-HCC liver tissues were shown for the (**A**) GSE22058, (**B**) GSE25097, (**C**) GSE36376, (**D**) GSE63898, (**E**) GSE64041 and (**F**) TCGA(LIHC) datasets (unpaired t-test). SRD5A3 expression in HCC tumor tissues and adjacent non-HCC liver tissues (peri-HCC) were shown for the (**G**) GSE54236, (**H**) GSE64041 and (**I**) TCGA(LIHC) datasets ((unpaired t-test)). SRD5A3 mRNA level was shown as log2 copy number units. LIHC, liver hepatocellular carcinoma.

**Figure 3:**
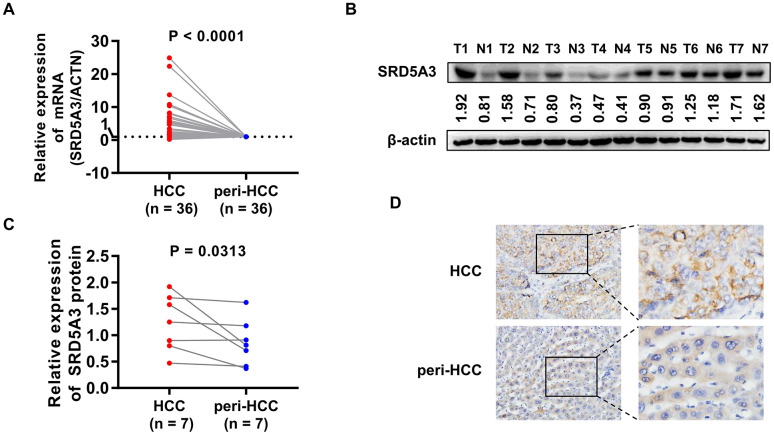
**qRT-PCR, Western blot and IHC measurements of SRD5A3 in HCC and peri-HCC tissues.** (**A**) The mRNA expression of SRD5A3 in 36 pairs of HCC and peri-HCC tissues was measured by qRT-PCR analysis. The SRD5A3 mRNA levels of peri-HCC tissues were normalized as ‘1’. (**B**, **C**) The protein level of SRD5A3 in 7 pairs of HCC and peri-HCC tissues was analyzed by Western blot. (**D**) The location of SRD5A3 protein was detected by IHC staining. T, HCC tumor tissue; N, peri-HCC tumor tissue.

We first employed the UALCAN web portal to explore the general expression of SRD5A3 in different cancers (Figure 1). Various cancers including HCC showed moderate to strong expression of SRD5A3 compared to their corresponding normal tissues. In order to verify whether this high expression of SRD5A3 in HCC tumor tissues is a common phenomenon, seven HCC datasets including GSE22058, GSE25097, GSE36376, GSE63898, GSE64041, TCGA(LIHC), and GSE54236 were analyzed (Figure 2). Bioinformatics analysis of these datasets revealed that the expression of SRD5A3 in HCC tissues was generally higher than that in non-HCC liver tissues (Figure 2A–2F) and corresponding adjacent liver tissues (Figure 2G–2I). Additionally, we performed quantitative reverse-transcription polymerase chain reaction (qRT-PCR) and Western blot analyses to verify this speculation again. Our data displayed that the mRNA and protein expression of SRD5A3 were significantly increased in the tumor tissues of 36 HCC patients compared with the matched adjacent non-HCC liver tissues (Figure 3A–3C), which was consistent with the results in public datasets. Immunohistochemistry (IHC) staining showed that SRD5A3 protein was predominantly located in the cytoplasm of HCC and liver cells (Figure 3D).

### Up-regulation of SRD5A3 is associated with the tumor progression of HCC

**Figure 4:**
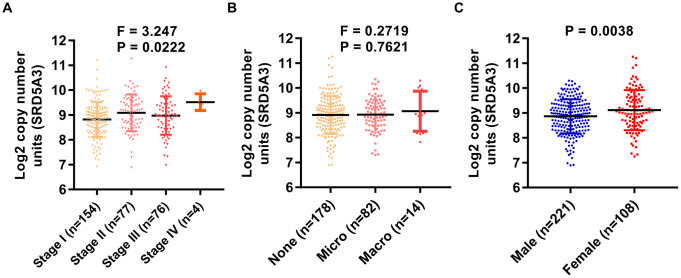
**The expression of SRD5A3 is correlated with the clinical stage and the gender of HCC patients.** In the TCGA(LIHC) dataset, the correlation between (**A**) clinical stage, (**B**) vascular invasion, (**C**) gender, and SRD5A3 expression was analyzed in HCC patients with clinical data. LIHC, liver hepatocellular carcinoma (HCC).

Next, the association between SRD5A3 expression and corresponding clinicopathological characteristics was evaluated. We found that the expression of SRD5A3 had a significant correlation with the clinical stage of HCC (P = 0.0222, F = 3.247, Figure 4A). Moreover, as vascular invasion intensified, the average expression of SRD5A3 seemed to increase; however, no significant statistical difference was detected among them (Figure 4B). Interestingly, we found that the expression of SRD5A3 was also associated with the patient's gender (Figure 4C). The average expression of SRD5A3 in female HCC patients tended to be higher than that of male HCC patients (Figure 4C).

### Up-regulation of SRD5A3 is associated with the prognosis of cancers including HCC

**Figure 5:**
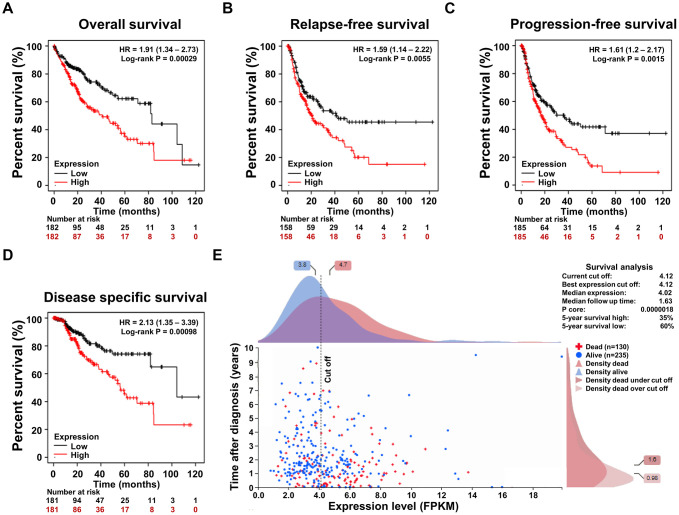
**The association of the SRD5A3 expression and the prognosis of HCC.** (**A**) Overall survival (OS), (**B**) relapse-free survival (RFS), (**C**) progression-free survival (PFS) and (**D**) disease specific survival (DSS) curve based on high and low expression levels of SRD5A3 from the Kaplan–Meier plotter survival analysis platform. (**E**) Prognostic significance of SRD5A3 expression in HCC in the Human Protein Atlas database. HR, hazard ratio.

To confirm the correlation between the SRD5A3 expression and the prognosis of HCCs, survival rates between the high and low SRD5A3 level groups were compared. We performed these analyses on the Kaplan–Meier plotter survival analysis platform, and found that HCC patients in the high SRD5A3 expression group had poorer overall survival (OS, HR = 1.91 (1.34 – 2.73), log-rank test P = 0.00029), relapse-free survival (RFS, HR = 1.59 (1.14 – 2.22), log-rank test P = 0.0055), progression-free survival (PFS, HR = 1.61 (1.20 – 2.17), log-rank test P = 0.0015) and disease specific survival (DSS, HR = 2.13 (1.35 – 3.39), log-rank test P = 0.00098) rates than that in the low SRD5A3 expression group (Figure 5A–5D). These were consistent with the data of HCC in the Human Protein Atlas database (Figure 5E), which again indicated that up-regulation of SRD5A3 expression predicts poor prognosis of HCC.

**Figure 6:**
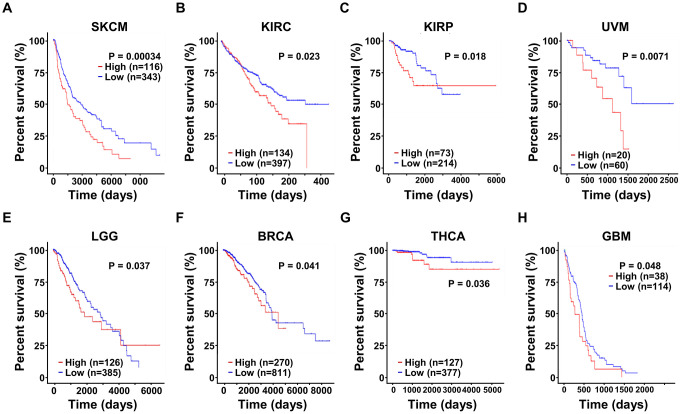
**The expression of SRD5A3 has prognostic value in various malignant cancers.** In the UALCAN website portal, the effect of high and low expression levels of SRD5A3 on the overall survival of (**A**) skin cutaneous melanoma (SKCM), (**B**) kidney renal clear cell carcinoma (KIRC), (**C**) kidney renal papillary cell carcinoma (KIRP), (**D**) uveal melanoma (UVM), (**E**) brain lower grade glioma (LGG), (**F**) breast invasive carcinoma (BRCA), (**G**) thyroid carcinoma (THCA) and (**H**) glioblastoma multiforme (GBM) cancer patients.

Moreover, we also explored the prognostic significance of SRD5A3 in other cancers, and found that the high expression of SRD5A3 was associated with the poor OS rates of skin cutaneous melanoma (SKCM, P = 0.00034), kidney renal clear cell carcinoma (KIRC, P = 0.023), kidney renal papillary cell carcinoma (KIRP, P = 0.018), uveal melanoma (UVM, P = 0.0071), brain lower grade glioma (LGG, P = 0.037), breast invasive carcinoma (BRCA, P = 0.041), thyroid carcinoma (THCA, P = 0.036), and glioblastoma multiforme (GBM, P = 0.048) (Figure 6A–6H). These results suggested that high expression of SRD5A3 may be a common unfavorable prognostic factor for many different types of cancer.

### Knockdown of SRD5A3 suppresses the growth of HCC *in vitro* and *in vivo*

**Figure 7:**
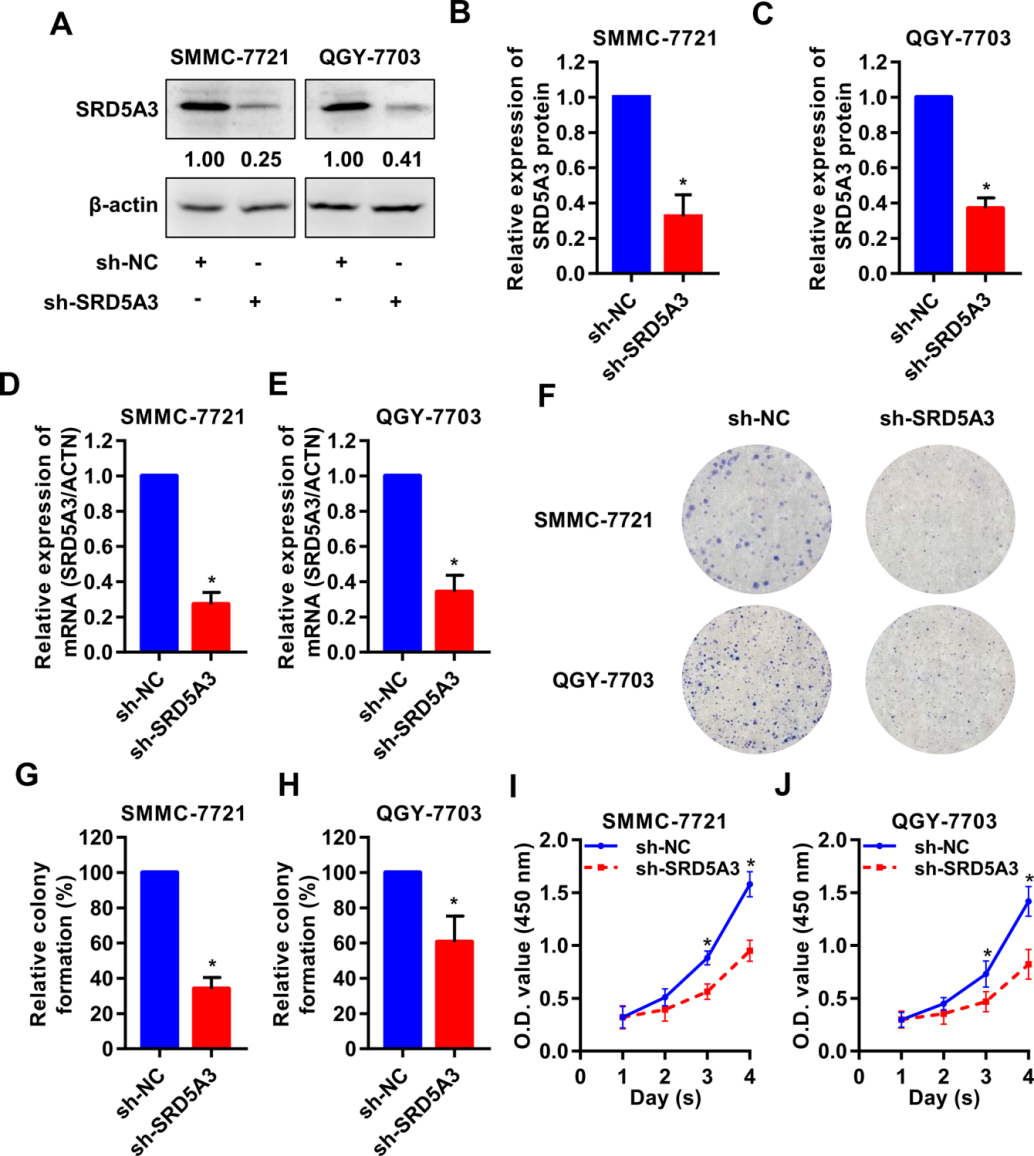
**SRD5A3 inhibition suppresses cell proliferation of HCC *in vitro*. SMMC-7721 and QGY-7703 cell lines were infected with sh-SRD5A3 or sh-NC lentivirus.** The knockdown efficiency of SRD5A3 was confirmed by (**A**–**C**) Western blot and (**D**, **E**) qRT-PCR analyses. (**F**–**H**) Colony formation assays were performed and analyzed. (**I**, **J**) CCK-8 assays were conducted to measure cell viability (two-way ANOVA analysis). NC, negative control. *, P value < 0.05.

**Figure 8:**
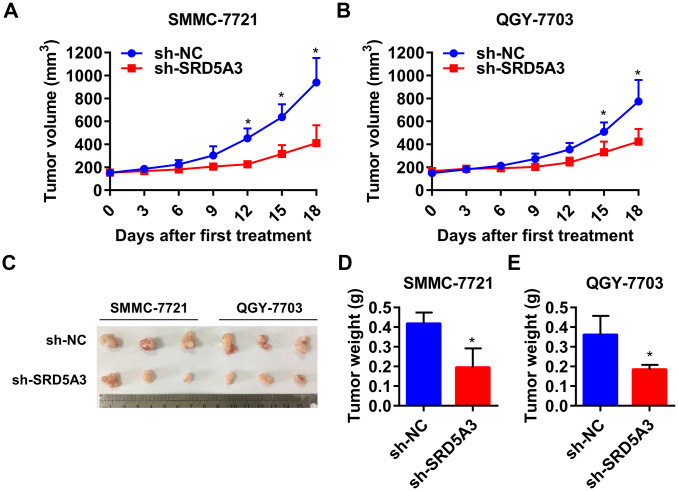
**Down-regulation of SRD5A3 suppresses HCC cell growth *in vivo*.** (**A**, **B**) Growth curve of the xenograft tumors (two-way ANOVA analysis). (**C**, **D**) Images and weight of xenograft tumors in sh-SRD5A3 and sh-NC groups (unpaired t-test). Three mice per group. NC, negative control. Error bar, mean with standard deviation. *, P value < 0.05.

To investigate the potential biological function of SRD5A3 in HCC tumorigenesis, we knocked down the expression level of SRD5A3 in HCC cell lines by infecting them with SRD5A3 shRNA (sh-SRD5A3) lentivirus or control vector (sh-NC). The knockdown efficiency was evaluated by Western blot and qRT-PCR analyses (Figure 7A–7E). Colony formation and cell viability assays were applied to reflect the proliferation inhibitory ability in the HCC cells lacking SRD5A3. As shown in Figure 7F–7J, the colony formation rate and the cell viability of HCC cell lines infected with sh-SRD5A3 were lower than that of cells infected with sh-NC. Furthermore, we inoculated HCC cells infected with sh-SRD5A3 or sh-NC in immunodeficient mice subcutaneously. The tumor growth curve, image and weight suggested that the down-regulation of SRD5A3 inhibited the growth of HCC (Figure 8A–8E).

### Molecular mechanisms behind the cellular function of SRD5A3

**Figure 9:**
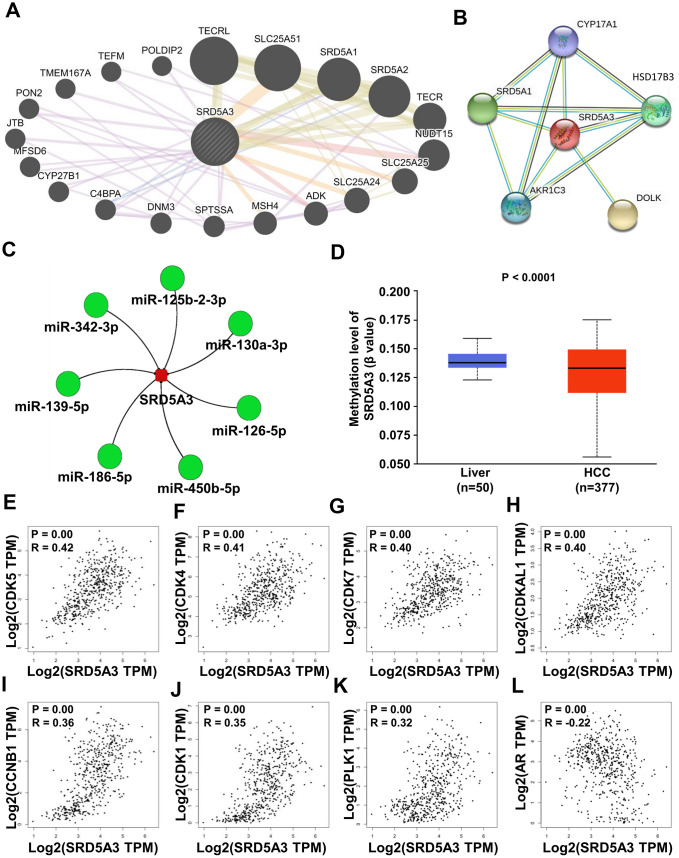
**Potential molecular mechanisms behind the effects of SRD5A3.** (**A**, **B**) Protein-protein interaction network between SRD5A3 and other protein-coding genes from the GeneMANIA and STRING databases. (**C**) Upstream microRNA of SRD5A3 from the miRNACancerMAP web-portal. (**D**) Methylation level of SRD5A3 from the UALCAN web portal. (**E**–**L**) Correlation analysis between CDK5, CDK4, CDK7, CDKAL1, CCNB1, CDK1, PLK1, AR and SRD5A3 in the GEPIA2 website portal. R, correlation coefficient.

Finally, the potential molecular mechanisms behind the effects of SRD5A3 in HCC were predicted. The GeneMANIA and STRING databases showed that SRD5A3 might interact with TECRL, SLC25A51, SRD5A1, SRD5A2, TECR, NUDT15, SLC25A25, SLC25A24, ADK, MSH4, SPTSSA, DNM3, C4BPA, CYP27B1, MFSD6, JTB, PON2, TMEM167A, TEFM, POLDIP2, SYP17A1, HSD17B3, DOLK, AKR1C3, SRD5A1 and etc. (Figure 9A, 9B). Moreover, the miRNACancerMAP web portal predicted several more reliable microRNAs upstream of SRD5A3 based on the TCGA(LIHC) dataset. These microRNAs included miR-125b-2-3p, miR-130a-3p, miR-126-5p, miR-450b-5p, miR-186-5p, miR-139-5p and miR-342-3p (Figure 9C). And the UALCAN web portal showed that SRD5A3 was hypomethylated in HCC tissues (Figure 9D), suggesting that the high level of SRD5A3 in HCC might be related to the hypomethylation status. Moreover, the GEPIA2 web portal showed that SRD5A3 was positively correlated with some genes such as PPIAP22 (R=0.56, P<0.05), EIF3K (R=0.56, P<0.05), KIAA1522 (R=0.54, P<0.05), MYL6 (R=0.54, P<0.05), PPIA (R=0.53, P<0.05), MRPS12 (R=0.53, P<0.05), SUMF2 (R=0.53, P<0.05), RRP36 (R=0.53, P<0.05), ORMDL2 (R=0.53, P<0.05) and etc. (Table 1). Some of them were genes related to the cell cycle, including CDK5 (R=0.42, P<0.05), CDK4 (R=0.41, P<0.05), CDK7 (R=0.40, P<0.05), CDKAL1 (R=0.40, P<0.05), CCNB1 (R=0.36, P<0.05), CDK1 (R=0.35, P<0.05) and PLK1 (R=0.32, P<0.05) (Figure 9E–9K and Table 1). Additionally, AR expression was found to have a negative correlation with SRD5A3 (Figure 9L and Table 1).

**Table 1:** Correlation between the expression of SRD5A3 and other genes.

**Gene symbol**	**Gene identification**	**Pearson's correlation coefficient (R value)**	**P value**
PPIAP22	ENSG00000198618.5	0.56	<0.05
EIF3K	ENSG00000178982.9	0.56	<0.05
KIAA1522	ENSG00000162522.10	0.54	<0.05
MYL6	ENSG00000092841.18	0.54	<0.05
PPIA	ENSG00000196262.13	0.53	<0.05
MRPS12	ENSG00000128626.11	0.53	<0.05
SUMF2	ENSG00000129103.17	0.53	<0.05
RRP36	ENSG00000124541.6	0.53	<0.05
ORMDL2	ENSG00000123353.9	0.53	<0.05
APOA1BP	ENSG00000163382.11	0.52	<0.05
SLC35E1	ENSG00000127526.13	0.52	<0.05
H2AFV	ENSG00000105968.18	0.52	<0.05
SLC35B2	ENSG00000157593.16	0.52	<0.05
COX5A	ENSG00000178741.11	0.52	<0.05
UQCR10	ENSG00000184076.12	0.52	<0.05
LSM2	ENSG00000204392.10	0.52	<0.05
MEA1	ENSG00000124733.3	0.51	<0.05
ATP5O	ENSG00000241837.6	0.51	<0.05
NOA1	ENSG00000084092.6	0.51	<0.05
CHMP3	ENSG00000115561.14	0.51	<0.05
RPN2	ENSG00000118705.16	0.51	<0.05
YWHAB	ENSG00000166913.12	0.51	<0.05
BOLA3	ENSG00000163170.11	0.51	<0.05
OST4	ENSG00000228474.5	0.51	<0.05
UFC1	ENSG00000143222.11	0.51	<0.05
ATG7	ENSG00000197548.12	0.48	<0.05
BAX	ENSG00000087088.19	0.47	<0.05
BAK1	ENSG00000030110.12	0.47	<0.05
CLOCK	ENSG00000134852.14	0.46	<0.05
CBX3	ENSG00000122565.18	0.46	<0.05
CDK5	ENSG00000164885.12	0.42	<0.05
CDK4	ENSG00000135446.16	0.41	<0.05
CDK7	ENSG00000134058.10	0.4	<0.05
CDKAL1	ENSG00000145996.11	0.4	<0.05
CCNB1	ENSG00000134057	0.36	<0.05
CDK1	ENSG00000170312	0.35	<0.05
PLK1	ENSG00000166851	0.32	<0.05
AR	ENSG00000169083	-0.22	<0.05

The statistics of these data are based on the GEPIA2 web portal.

## DISCUSSION

HCC is one of the most aggressive and enigmatic malignant tumors worldwide, causing millions of deaths per year [1]. The carcinogenesis and development of HCC are generally considered to be related to a variety of factors, such as hepatitis B or C virus infection, cirrhosis, fatty liver disease, diabetes, alcohol, aflatoxin, aristolochic acid and so on [2]. Although some exact genes and pathways driving the pathogenesis of HCC have been discovered, many others have not yet been detected and explored [18, 19].

In the human genome, SRD5A3 is located on chromosome 4 and is about 36 kDa in length. It is a member of the steroid 5-alpha reductase family which is considered to play an important role in male sexual development [8–10]. Here, we found SRD5A3 was generally upregulated in HCC as well as many other malignant tumor tissues. As is well-known, HCC is more prevalent in males than in females, with a gender-specific ratio of 2:1 to 8:1 [20, 21]. Males develop HCC faster and more severe than females [20]. The reason for this gender disparity is related to sex hormones, but it is not well understood yet [22].

Recent studies indicated that the activation of androgen-AR pathway could accelerate the pathogenesis and progression of HCC [22–24]. The expression of AR was discovered to be elevated in HCC tissues compared to that in non-HCC liver tissues [22–24]. And the overexpression of AR promoted hepatocarcinogenesis and cell growth of HCC via promoting DNA damage and reactive oxidative stress, as well as suppressing DNA damage sensing and repairing system mediated by p53 [23]. In addition, mouse liver lacking AR was found to develop later and less tumorigenesis than the normal liver [23]. These results suggested that targeting androgen-AR signaling was a potential strategy for HCC therapy [22–24].

The maintenance of androgen-AR activation pathway requires steroid 5-alpha reductase enzymes (SRD5A1, SRD5A2, and SRD5A3), and this signaling pathway also regulates the mRNA expression of these steroid 5-alpha reductase enzymes [8–10]. Thus, steroid 5-alpha reductase enzymes, such as SRD5A3, may be related to the gender disparity in patients with HCC and affect the progression of HCC. Previously, SRD5A3 was confirmed to be overexpressed in human fetal liver [17]. The gender differences existed in the expression of SRD5A3 in fetal liver (higher in males) [17]. Interestingly, in the present study, we also found a significant difference between the expression of SRD5A3 in male and female HCCs, while the SRD5A3 expression was negatively correlated with the AR expression. These data hinted SRD5A3 might be involved in the regulation of androgen-AR signaling. However, unlike expected, the SRD5A3 expression in female HCC tissues was higher than that in males. The reason was not clear and needs further investigation. Moreover, our results showed that SRD5A3 was a malignant aggressive molecule and a poor prognostic factor in HCC patients. The suppression of SRD5A3 inhibited the HCC growth *in vitro* and *in vivo*, suggesting that SRD5A3 may also be a novel potential target for HCC therapy.

To investigate the mechanisms by which SRD5A1 interferes with HCC proliferation, we resorted to the interaction databases. Through the interaction analysis, multiple protein-coding genes and microRNAs were suggested to be related to SRD5A3. Among them, SRD5A1 [11, 12], SRD5A2 [13, 14], adenosine kinase (ADK) [25], Dynamin 3 (DNM3) [26], miR-125b-2-3p [27], miR-130a-3p [28], miR-126-5p [29], miR-450b-5p [30], miR-186-5p [31], miR-139-5p [32] and miR-342-3p [33] have been reported to influence the growth, invasion, metastasis or drug resistance of HCC. Furthermore, correlation exploration in the present study hinted that SRD5A3, together with other members of steroid 5-alpha reductase enzymes (SRD5A1 and SRD5A2), may also be correlated with other molecules, such as PPIAP22, EIF3K, KIAA1522 and etc. Moreover, the effect of SRD5A3 on HCC growth may involve the regulation of the cell cycle through CDK5, CDK4, CDK7, CDKAL1, CCNB1, CDK1, and PLK1.

Taken together, our bioinformatics analyses suggested that elevated SRD5A3 expression is associated with a depressing prognosis of various malignancies including HCC. The expression of SRD5A3 in female HCC tissues tends to be higher than that in males. Loss-of-function experiments indicated that SRD5A3 accelerates the development of HCC by promoting cell proliferation.

To the best of our knowledge, this is the first research to provide evidence of SRD5A3 as an oncogene by exploring its prognostic value, cellular function, and molecular mechanism in HCC. However, the sample size of HCC patients recruited from our affiliation was relatively small and the mechanism exploration was still only in the prediction stage. Therefore, further investigations are wanted in the follow-up studies.

## MATERIALS AND METHODS

### Bioinformatics analysis

SRD5A3 expression in various cancers and its relationship with tumor progression were analyzed via employing the UALCAN web portal (http://ualcan.path.uab.edu/index.html), the Gene Expression Omnibus (GEO) database (https://www.ncbi.nlm.nih.gov/geo/) and The Cancer Genome Atlas database (TCGA, https://cancergenome.nih.gov/). The impact of SRD5A3 expression on survival prognosis of different cancer patients was predicted by the Kaplan–Meier plotter survival analysis platform (http://kmplot.com/analysis/), Human Protein Atlas database (https://www.proteinatlas.org/) and UALCAN web portal. Methylation level of SRD5A3 was analyzed by UALCAN web portal. Potential interactions between SRD5A3 and other coding genes or microRNAs were predicted by using GeneMANIA (http://genemania.org/search/), STRING (https://string-db.org/cgi/input.pl), and miRNACancerMAP (http://cis.hku.hk/miRNACancerMAP/MAPQuest1.php) and GEPIA2 (http://gepia2.cancer-pku.cn/) website portals.

### Clinical specimens

HCC tumor (HCC) and adjacent non-HCC liver tissues (peri-HCC) were obtained from 36 patients who underwent hepatectomy after signing informed consent. The specimens were immediately stored in liquid nitrogen until analysis. The clinical data and pathological characteristics of these HCC patients were summarized in Supplementary Table 1. The application of HCC specimens was approved by the Ethics Committee of Guangdong Provincial People’s Hospital.

### RNA isolation and qRT-PCR

Total RNA was extracted from HCC tumor tissues and corresponding adjacent non-HCC liver tissues following the instruction of TRIzol reagent (Cat. no. T9109, TaKaRa, USA). The reverse transcription of mRNA was performed by using PrimeScript RT Master Mix Kit (Cat. no. RR036A, TaKaRa, USA). The cDNA generated above was used as a template to detect the mRNA expression of SRD5A3 with TB Green™ Premix Ex Taq™ detection kit (Cat. no. RR420A, TaKaRa, USA). β-actin gene acts as an internal control. Primer sequences were as follows: SRD5A3 (forward primer: 5′-TTTAATCAGGCCCTGTCTGC-3′, reverse primer: 5′-GGGGTATAGAAATGGAATGGAGA-3′); β-actin (forward primer: AGCGAGCATCCCCCAAAGTT, reverse primer: GGGCACGAAGGCTCATCATT). These primers were synthesized by RuiBiotech (China). The relative expression of SRD5A3 mRNA level was computed according to the 2^-ΔΔCт^ method.

### Western blot analysis

Total protein was extracted from specimens or cell lines using RIPA lysis buffer (Cat. no. #FD008, Fdbio Science, China) supplemented with protease inhibitor cocktail (Cat. no. #FD1001, Fdbio Science, China) and phosphatase inhibitor cocktail (Cat. no. #FD1002, Fdbio Science, China). The protein supernatant was collected after centrifuging at 14000 g and 4° C. Then, the concentration of the protein supernatant was quantified using bicinchoninic acid assay kit (Cat. no. #FD2001, Fdbio Science, China), and boiled at 100° C for 10 minutes. Equal amounts of protein from different samples were separated by polyacrylamide gel electrophoresis and transferred onto 0.45 μm nitrocellulose filter membranes (GE Healthcare, United States). Subsequently, the membranes were probed with primary antibodies against human SRD5A3 (Cat. no. #NBP1-69612, Novus, United States) or β-actin (Cat. no. #4970, Cell Signaling Technology, United States) overnight at 4° C, followed by incubation with a secondary horseradish peroxidase (HRP)-conjugated antibody for 60 minutes at room temperature. Finally, protein blot images were detected using an enhanced chemiluminescence kit (Cat. no. #WBKLS0100, Millipore, United States), and were quantified by using ImageJ software (Ver. 1.46r; MD, United States).

### Immunohistochemistry (IHC) staining

HCC tumor and adjacent non-HCC liver tissues embedded in paraffin were sectioned onto 3-μm-thick slices. After retrieving antigen by heating in citrate buffer (pH 6.0) and blocking endogenous peroxidase activity by H2O2, the slices were incubated with the primary antibody against SRD5A3 (Cat. no. #HPA027006, Sigma, United States) at a concentration of 1:50 overnight. Then, the slices were incubated with a secondary HRP-conjugated antibody. Subsequently, the slices were stained with diaminobenzidine (DAB) and counterstained with hematoxylin. Finally, the staining images were captured.

### Cell culture

Human HCC cell lines QGY-7703 and SMMC-7721 were obtained from the Guangzhou Jennio Biotech Co.,Ltd. (GD, China). Cells were maintained in high glucose Dulbecco’ s modified Eagle’ s medium (DMEM, Gibco, MA, United States) supplemented with 10 % fetal bovine serum (FBS, Gibco, MA, United States) at 37° C in 5% CO2 condition, and were routinely checked for mycoplasma contamination.

### Establishment of HCC cell lines with down-regulated SRD5A3

A lentiviral SRD5A3 interference vector was obtained from Hanbio Biotechnology Co., Ltd. (Shanghai, China). The sequence of SRD5A3 and negative control (NC) shRNA were as follows: AAATAAAGCAGGAGTGGTCATTC (sh-SRD5A3), TTCTCCGAACGTGTCACGT (sh-NC). Lentivirus production, viral infection and qRT-PCR were performed according to the manufacturer’s instructions. Positive and stable infectants were selected by puromycin.

### Colony formation

Colony formation experiment was performed as previously described [34, 35]. In brief, 500 cells infected with sh-SRD5A3 or sh-NC were seeded in 6-well plates. After culturing in DMEM medium supplemented with 10% FBS for 7 to 14 days, the cell colonies were fixed with methanol for 2 minutes, then stained with crystal violet for 30 minutes at room temperature. After washing away excess dye, the number of cell colonies was counted by imageJ (Ver. 1.46r; MD, United States) software.

### Cell viability measurement

3 × 103 cells were seeded per well in 96-well plate, then the CCK-8 (Dojindo, Japan) assay was performed every 24 hours for 4 consecutive days as previously described to measure cell viability [34, 36].

### Tumor xenograft experiments

BALB/c female nude mice (four-week-old) were subcutaneously inoculated with 5×10^6^ HCC cells stably infected with sh-SRD5A3 or sh-NC. Tumor length and tumor width were measured every 3 days for a total of 18 days. The tumor volume was calculated as length × width^2^/2. The animal experiment was approved by the Animal Health and Ethics Committee. All procedures for this experiment were conducted in accordance with the guidelines for the care and use of laboratory animals.

### Statistical analysis

All data were expressed as the mean ± standard deviation. Statistical comparisons were performed using unpaired or paired Student's t test in two-groups, or ANOVA (one-way or two-way) test among more than two groups. The OS, RFS, PFS and DSS survival curves of HCC patients were generated by Kaplan-Meier method, then analyzed via log-rank test. The analyses in this study were conducted through GraphPad Prism 6.0 software (United States). P value < 0.05 was defined as statistically significant.

## Supplementary Material

Supplementary Table 1
