# Supplementary material for: Steroid 5 alpha-reductase 3 (SRD5A3) promotes tumor growth and predicts poor survival of human hepatocellular carcinoma (HCC)
Source: Aging (Albany NY). 2020 Nov 20;12(24):25395–411. doi: 10.18632/aging.104142 (PMC7803539; doi:10.18632/aging.104142)
Supplement: Supplementary Table 1 [file aging-12-104142-s001.pdf]

SUPPLEMENTARY TABLE

Supplementary Table 1. Clinical features of 36 patients with HCC.

Variable	Number/Mean±SD
Age (year)	52.61 ± 12.59
Gender (male/female)	32/4
Family history of HCC (+/-)	5/31
History of drinking (+/-)	6/30
Cirrhosis (+/-)	27/9
HBV DNA (≥/<100 copies/ml)	15/21
HBsAg (+/-)	30/6
HBsAb (+/-)	4/32
HBcAb (+/-)	36/0
HBeAg (+/-)	2/34
HBeAb (+/-)	4/32
alpha-Fetoproteins (>20/≤20 μg/L)	18/18
Alanine aminotransferase (>40/≤40 U/L)	15/21
Aspartate aminotransferase (>40/≤40 U/L)	20/16
Albumin (>35/≤35 g/L)	22/14
Total bilirubin (>17/≤17 μmol/L)	14/22
γ-glutamyl transpeptidase (>100/≤100 U/L)	27/9
Tumor size (>3/≤3 cm)	28/8
Number of tumor nodules (>1/≤1)	7/29
Portal vein thrombus (+/-)	5/31
Distance metastasis (+/-)	0/36
Pathological differentiation (high/moderate/low)	1/15/20
TNM stage (I/II/III/IIIa/IIIb)	8/4/19/5

SD, standard deviation.
